# Validation of an Automated Quantitative Digital Pathology Approach for Scoring TMEM: A Prognostic Biomarker for Metastasis

**DOI:** 10.3390/cancers12040846

**Published:** 2020-03-31

**Authors:** David Entenberg, Maja H. Oktay, Timothy D’Alfonso, Paula S. Ginter, Brian D. Robinson, Xiaonan Xue, Thomas E. Rohan, Joseph A. Sparano, Joan G. Jones, John S. Condeelis

**Affiliations:** 1 Department of Anatomy and Structural Biology, Albert Einstein College of Medicine/Montefiore Medical Center, Bronx, NY 10461, USA; maja.oktay@einsteinmed.org (M.H.O.); joan.jones@einsteinmed.org (J.G.J.); 2 Gruss-Lipper Biophotonics Center, Albert Einstein College of Medicine/Montefiore Medical Center, Bronx, NY 10461, USA; 3 Integrated Imaging Program, Albert Einstein College of Medicine/Montefiore Medical Center, Bronx, NY 10461, USA; 4 Department of Pathology, Albert Einstein College of Medicine/Montefiore Medical Center, Bronx, NY 10461, USA; 5 Department of Pathology, Memorial Sloan Kettering Cancer Center, New York, NY 10021, USA; dalfonst@mskcc.org; 6 Department of Pathology and Laboratory Medicine, Weill Cornell Medicine, New York, NY 10065, USA; psg9003@med.cornell.edu (P.S.G.); brr2006@med.cornell.edu (B.D.R.); 7 Department of Epidemiology and Population Health, Albert Einstein College of Medicine/Montefiore Medical Center, Bronx, NY 10461, USA; Xiaonan.Xue@einsteinmed.org (X.X.); Thomas.Rohan@einsteinmed.org (T.E.R.); 8 Department of Oncology, Albert Einstein College of Medicine/Montefiore Medical Center, Bronx, NY 10461, USA; jsparano@montefiore.org; 9 Department of Surgery, Albert Einstein College of Medicine/Montefiore Medical Center, Bronx, NY 10461, USA

**Keywords:** TMEM, metastasis, prognostic, digital pathology, validation study

## Abstract

Metastasis causes ~90% of breast cancer mortality. However, standard prognostic tests based mostly on proliferation genes do not measure metastatic potential. Tumor MicroEnvironment of Metastasis (TMEM), an immunohistochemical biomarker for doorways on blood vessels that support tumor cell dissemination is prognostic for metastatic outcome in breast cancer patients. Studies quantifying TMEM doorways have involved manual scoring by pathologists utilizing static digital microscopy: a labor-intensive process unsuitable for use in clinical practice. We report here a validation study evaluating a new quantitative digital pathology (QDP) tool (TMEM-DP) for identification and quantification of TMEM doorways that closely mimics pathologists’ workflow and reduces pathologists’ variability to levels suitable for use in a clinical setting. Blinded to outcome, QDP was applied to a nested case-control study consisting of 259 matched case-control pairs. Sixty subjects of these were manually scored by five pathologists, digitally recorded using whole slide imaging (WSI), and then used for algorithm development and optimization. Validation was performed on the remainder of the cohort. TMEM-DP shows excellent reproducibility and concordance and reduces pathologist time from ~60 min to ~5 min per case. Concordance between manual scoring and TMEM-DP was found to be >0.79. These results show that TMEM-DP is capable of accurately identifying and scoring TMEM doorways (also known as MetaSite score) equivalent to pathologists.

## 1. Introduction

Breast cancer is a major public health problem resulting in approximately 520,000 deaths worldwide each year [1]. The overwhelming cause of mortality from breast cancer is due to distant metastases which can occur even after long surveillance periods. In recent years, a number of clinical biomarkers (primarily based on measuring cell proliferation and estrogen regulation) that offer prognostic information regarding recurrence have come to market (e.g., Oncotype DX and MammaPrint) [2,3]. While the clinical utility of this information is unquestionable, these biomarkers do not provide conclusive evidence about the systemic dissemination of tumor cells and the long-term risk of distant metastatic disease for all patients. 

A new metric, termed Tumor MicroEnvironment of Metastasis (TMEM) [4], also known as MetaSite Score [5], has recently been shown to be prognostic of metastatic risk in estrogen receptor positive / human epidermal growth factor receptor 2 negative (ER^+^/HER2^−^) breast cancer, independent of the four-stain immunohistochemical assay (IHC4) [6], the genomic clinical recurrence biomarker (OncotypeDx score) [2], and classical clinicopathologic features [7] and was clinically validated [5,7]. TMEM is a microanatomical marker based upon preclinical multiphoton imaging studies [8,9,10,11] which showed 1) that intravasation of cancer cells occurs within the tumor mass at TMEM doorways where a tumor cell expressing high levels of the actin regulatory protein Mena [12], a macrophage, and an endothelial cell make stable physical contact and 2) that TMEM is the actual doorway for hematogenous dissemination of tumor cells [9,11,13,14]. 

Before the development of the digital pathology-based TMEM doorway scoring algorithm described here (TMEM-DP), TMEM doorway quantification required manual identification and scoring by pathologists using triply stained formalin-fixed paraffin embedded (FFPE) archival tissue sections. Identification involved looking for direct contact of the three cell types that compose TMEM doorway within tumor nests. As described below, this task is labor intensive, requiring the pathologist to scan the slide at low power, to identify potential regions of interest, to switch to high power (40× magnification), to digitally capture 10 high-power fields of view, and to finally score the images. The entire process has been timed to take approximately 1 hour per case. 

While the use of digital images to aid in pathologic diagnosis is not new, the relatively recent technological development of whole slide imaging (WSI) holds the promise to dramatically expand the capabilities of pathology laboratories with applications in telepathology [15], education [16], quantification of biomarkers (in both immunohistochemistry [IHC] and fluorescence) [17,18,19], as well as reduction of pathologist fatigue and error rate via standardization of methodologies. Numerous preliminary studies have shown excellent concordance between traditional and digital pathology [20,21,22]. 

The present study was undertaken to evaluate the performance of a digital pathology-based identification and scoring method for TMEM doorway quantification that replicates the pathologists’ workflow, maintains or improves reproducibility, and dramatically reduces pathologist time required. We show that the TMEM-DP algorithm is capable of accurately identifying and scoring TMEM doorways nearly as well as pathologists while reducing pathologist time from ~1 h to ~5 min per case. This validation study is crucial to establishing the feasibility of utilizing digital pathology for the semiautomated quantification of biomarkers of metastasis and can serve as a template for future such studies.

## 2. Results

### 2.1. Algorithm Development

**Figure 1:**
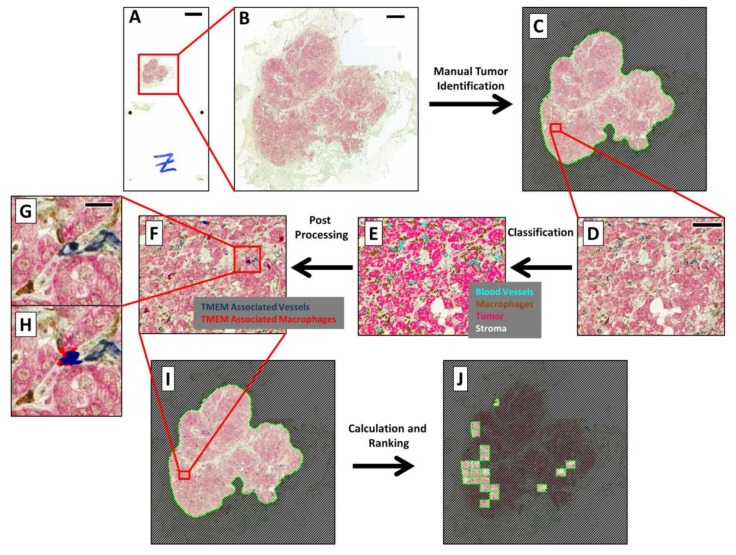
Algorithm for performing automated Tumor MicroEnvironment of Metastasis (TMEM) doorway analysis and scoring on whole scanned slides: Automated identification of TMEM doorway in immunohistochemical(IHC)-stained slides requires several steps applied at different magnifications. (**A**) Digitized whole slide captured on the 3DHistech Pannoramic P250 slide scanner. Bar = 5000 µm. (**B**) Zoomed-in region of just the tissue. Bar = 1000 µm. (**C**) Pathologist identification of invasive tumor by drawing a region of interest (ROI) separating and masking off the tumor tissue from the surrounding stroma. (**D**) A 40× equivalent zoomed-in field-of-view within the tumor area. Bar = 100 µm. (**E**) Classification of each pixel as either a blood vessel (cyan), a macrophage (brown), a tumor cell (pink), or stroma (uncolored). (**F**) Identification of TMEM doorways highlights the TMEM-associated vessels in dark blue and the TMEM-associated macrophages in red. (**G**) Zoomed-in view of one identified TMEM doorway from Figure 1F. Bar = 20 µm. (**H**) Same as Figure 1G with the overlay indicating the TMEM-associated vessel (dark blue) and TMEM-associated macrophage (red). (**I**) Image of the whole tissue after application of both the tumor identification and TMEM identification algorithms. (**J**) The top 10 scoring fields are highlighted by the TMEM ranking algorithm.

Crucial to the success of this study was the development of a digital pathology-based algorithm for TMEM doorway identification. Due to the high level of intratumoral heterogeneity present in clinical breast cancer samples, WSI was employed as it allows identification of individual cell types across the entire tumor tissue and thus enabled TMEM doorway quantitation within regions of the tumor with their highest density. An example digital whole slide is shown in Figure 1A along with a zoom-in to just the tissue area (Figure 1B). 

Image analysis was performed using a custom app developed in Visiopharm’s VisiomorphDP software. An example of the procedure is illustrated in Figure 1C. Image analysis was performed only on regions of invasive tumor predefined by the pathologists. Identification involved creating Regions of Interest (ROIs) in VisiomorphDP (Figure 1C) which required only 1–2 min per case of pathologist time.

**Figure 2:**
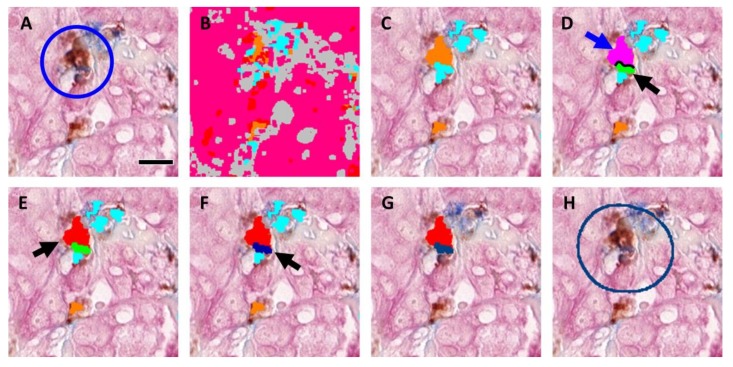
Morphometric analysis and identification of TMEM doorways: (**A**) A representative field of view containing one TMEM doorway (circled). Tissue is stained for Pan-Mena-overexpressing tumor cells (pink), macrophages (brown), and endothelial cells (blue). Bar = 15 µm (**B**) A linear Bayesian classifier identifies each pixel as belonging to one of five different categories: blood vessels (cyan), macrophages (brown), lightly stained tumor cells (pink), darkly stained tumor cells (red), or background and stroma (gray). (**C**) Contiguous groups of pixels with the same class are treated as objects which are then subjected to image processing steps to smooth boundaries and filter of small and large objects, leaving macrophages (orange), vessels (cyan), tumor (label not shown in figure), and stroma/background (label not shown in figure). (**D**) Morphometric analysis allows the reclassification of macrophages and vessels as macrophage–vessel complexes (magenta, blue arrow) and vessel–macrophage complexes (green, black arrow) according to their degree of boundary contact (black line). (**E**,**F**) Further morphometric analysis identifies macrophage–vessel complexes and vessel–macrophage complexes with prespecified boundary contacts with tumor cells as TMEM-associated macrophages (red) and TMEM-associated vessels (dark blue), respectively. (**G**) TMEM-associate macrophages and TMEM-associated vessels are isolated from all other structures and considered TMEM objects. (**H**) TMEM objects are then marked by expanding the size of the TMEM-associated vessels by 140 pixels (50 µm) to generate a circle resembling those utilized by the pathologists during manual scoring.

As with all apps in VisiomorphDP, analysis was split into five distinct steps:

1. Preprocessing—RGB images, captured with the slide scanner’s color camera, were split into “Feature Channels”, which optimized the signal contrast for each of the three TMEM stains utilized. A median filter was applied at this stage to aid in the signal separation.
2. Classification—A linear Bayesian classifier algorithm [23] was trained to identify and classify each pixel into one of five categories (macrophages, darkly stained tumor cells, lightly stained tumor cells, blood vessels, and stroma) by pathologist-guided identification of each of the stains within fields of view extracted from 6 different slides. Two categories were utilized for the tumor cells to accommodate variations in staining. Training was performed during the development of the algorithm and remained unchanged thereafter. The classifier algorithm was then applied to the slide one high-power field at a time. This is demonstrated in Figure 1D, which shows the IHC staining in a single high-power field of view, and in Figure 1E, which shows the same field with each pixel pseudo-colored by its classification.
3. Postprocessing—Contiguous groupings of pixels in each classification were treated as separate objects, and image analysis steps were next applied to manipulate and redefine these classified objects based upon size, morphometry, and spatial relations to other objects, ultimately resulting in the identification of TMEM objects (Figure 1H and Figure 2). After identification of TMEM objects, the entire digital slide was globally scored (Figure 1I) and ranked (Figure 1J) as described below.
4. The steps for identification of TMEM objects are demonstrated in Figure 2 using a field of view containing a single TMEM doorway (Figure 2A) (manually identified and marked by a circle). Figure 2B shows the same field of view after pixel classification by the trained algorithm. Next, the two classifications of tumor (pink and red) were merged and the boundaries of all objects (tumor, stroma (gray), vessels (cyan), and macrophages (orange)) were smoothed by enlarging (dilating) and subsequently shrinking (eroding) each object. In addition, large vessels and macrophage aggregates (>800 µm^2^) were also removed along with small fragments of tumor tissue (<10 µm^2^), stroma (<400 µm^2^), particles resembling macrophages (<5 µm^2^), and particles resembling vessels (<5 µm^2^). Figure 2C shows the macrophages (orange) and vessels (cyan) that were identified using this procedure. Objects were redefined based on cell–cell contacts as follows:
  a. Vessels that have at least 2% of their perimeter in contact with macrophages (Figure 2D, black line) were labeled as separate objects called “vessel–macrophage complexes” (Figure 2D, green object, black arrow).
  b. Macrophages that are in contact with vessels that have been identified as vessel–macrophage complexes were labeled as separate objects called “macrophage–vessel complexes” (Figure 2D, magenta object, blue arrow).
  c. Macrophages that have been identified as macrophage–vessel complexes were evaluated for their amount of contact with Mena+ tumor cells and vessels depending upon their size. These contacts were defined as follows:
    - Large macrophage–vessel complexes (≥40 µm^2^) that have at least 8% of their perimeter in contact with tumor cells and at least 20% of their perimeter in contact with vessels were labeled as “large TMEM-associated macrophages” (Figure 2E, red object, black arrow),
    - Small macrophage–vessel complexes (<40 µm^2^) that have at least 10% of their perimeter in contact with tumor tissue and at least 10% of their perimeter in contact with vessels were labeled as “small TMEM-associated macrophages” (label not shown in figure),
  d. Vessels that have at least 20% of their perimeter in contact with tumor cells and at least 20% of their perimeter in contact with small and large TMEM-associated macrophages were labeled as “TMEM-associated vessels” (Figure 2F, dark blue object, black arrow),
  e. All objects other than small and large TMEM-associated macrophages and TMEM-associated vessels were removed (Figure 2G).

As described further below, “TMEM objects” were then created by enlarging the area around TMEM associated vessels by 140 pixels (50 µm) to simulate the circles utilized by the pathologists (Figure 2H, dark blue circle).

5. Calculation—Metrics were applied to the final classified pixel clusters to quantify the objects (e.g., object counts, object areas, and interface lengths).
6. Ranking—The entire tissue within pathologist’s defined region of interest (ROI) was divided into areas equivalent to a pathologist’s microscope high-power field (~300 × 400 µm, Figure 1I), and each area was individually analyzed. The TMEM doorway count for each area was calculated by summing the number of identified TMEM objects within the area. The counts for all of the areas were then compared and ranked, and the highest scoring areas were finally presented to the pathologist for review (Figure 1J). The final TMEM doorway score for the entire patient sample was determined as the sum of the counts of the TMEM objects within the top 10 ranked fields of view.

### 2.2. Determination of Appropriate Metric for Automated Algorithm

After evaluation for its ability to accurately identify and quantify TMEM doorways, the commercially available software package VisiomorphDP was chosen for its relative ease of use and its ability to handle complex morphometric relationships. However, VisiomorphDP was unable to recreate the process of marking the juxtaposition of the three cells forming a TMEM doorway with circles, as is performed by the pathologists. As such, an alternative method of identification and enumeration of TMEM objects was needed. After identification of the cells composing a TMEM doorway, several alternatives were evaluated and directly compared (Table 1) including enlarging the area of the TMEM-associated vessel objects (Figure 2G, dark blue object), counting them, and calculating their sum; enlarging the area the TMEM-associated macrophages (Figure 2G, red object), counting them, and calculating their sum; and calculating the total length of the interface between TMEM-associated macrophages and TMEM-associated vessels (Figure 2G, boundary between red and dark blue objects). 

While all of the metrics highly correlated with each other (Spearman rank correlation > 0.96), and could have been utilized, the enlarged area of TMEM-associated vessels (Figure 2H, dark blue circle) produced scores that most closely mimicked the scale of the scores (~1 per underlying TMEM structure) used by the pathologists’ method and produced scores that were more intuitive. As such, the sum of TMEM-associated vessels was taken as the metric of choice.

### 2.3. Evaluation of Algorithm Performance

#### Pathologist Inter- and Intra-Observer Validation

**Figure 3:**
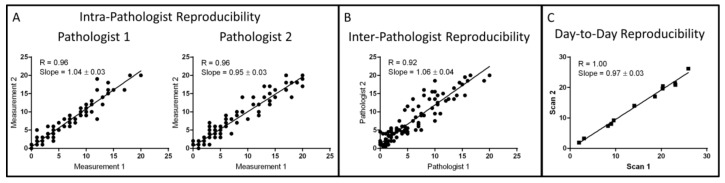
Summary of algorithm reproducibility tests: (**A**) Intra-pathologist reproducibility evaluated by comparison of TMEM scores from 100 different images manually generated by two different pathologists on two different days. (**B**) Inter-pathologist reproducibility evaluated by comparison of the average TMEM scores from Figure 3A for each pathologist. (**C**) Day-to-day reproducibility of the combined scanning algorithm performance using 12 cases scanned and evaluated on different days.

In order to evaluate the reproducibility of both the pathologists and the algorithm in identifying TMEM doorways, 100 representative fields of view from 22 cases were manually selected for scoring by both the algorithm and by two pathologists independently on two separate occasions. These fields contained an estimated total TMEM sum of greater than 600, with a range of TMEM (0–20) in each field of view. Intra-observer reproducibility was evaluated using a Pearson’s correlation coefficient. The “within observer” agreement was 0.96 for both pathologists (Figure 3A), and the “between observer” level of agreement (using the average of scores for each pathologist) was 0.92 (Figure 3B). 

A second test of the interobserver performance was done to evaluate the full pathologic analysis which included both field selection and TMEM scoring. In this case, the same 60 cases which were utilized to develop the algorithm were analyzed by five pathologists independently and agreement was assessed using a Pearson’s correlation. The average inter-pathologist correlation was determined to be R = 0.74.

### 2.4. Algorithm Reproducibility

Evaluation of the total system reproducibility was accomplished by scanning 12 different slides on two separate occasions and by evaluating their TMEM score with the automated algorithm. Near-perfect repeatability (R = 1.00) was verified using a Pearson’s correlation analysis (Figure 3C).

### 2.5. Algorithm Performance

**Figure 4:**
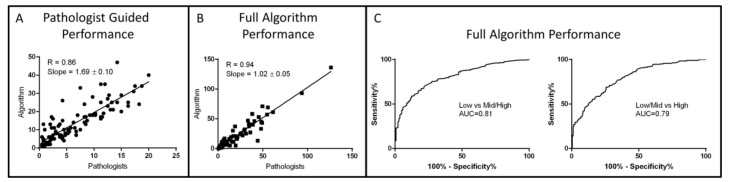
Summary of algorithm performance and validation tests: (**A**) Evaluation of algorithm’s performance in identifying TMEM doorways in the same images used in Figure 3A by comparison to the average pathologist scores. (**B**) Evaluation of algorithm’s performance in both identifying high-ranking TMEM fields and by scoring them by comparison with scores generated by five different pathologists using a cohort of 59 cases. (**C**) Performance of the algorithm in classifying cases into low-risk and high-risk categories as determined by the cutoff points of 6 (left) and 23 (right) TMEM.

Performance of the algorithm was evaluated in three different ways: First, the algorithm’s ability to score TMEM doorways (“pathologist guided analysis”) was evaluated by processing with the algorithm the same 100 fields of view that were utilized to assess the inter- and intra-pathologist reproducibility. For this analysis, algorithm scores were directly compared to the average of scores generated by the two pathologists independently. Correlation and concordance between the pathologists’ average scores and the algorithm generated scores were high with a Pearson R = 0.86 (Figure 4A) and area under the curve (AUC) of 0.90 and 0.96 when comparing tertiles (low vs mid/high scores and low/mid vs high scores). 

In the second method for performance evaluation, the algorithm’s ability to replicate the entire pathologic analysis, including field selection and TMEM scoring (“full algorithm analysis”), was evaluated on the 60 cases that were used to generate the algorithm and its scores were directly compared to the average of manual scoring by five pathologists. Correlation and concordance, after exclusion of one case which was more than ten standard errors from the regression line, was again extremely high with a Pearson’s R = 0.94 (Figure 4B) and an AUC of 0.95 and 0.94 when comparing tertiles (low vs mid/high scores and low/mid vs high scores). Receiver operating characteristic (ROC) analysis was also performed utilizing the low-risk and high-risk cutoff TMEM scores of 6 and 23, respectively, that were determined by Rohan et al. [7], giving AUC = 0.97 and 1.00 respectively.

Finally, in the third method, the performance of the full algorithm analysis was evaluated on the entire cohort of 468 evaluable cases (13 cases from the original cohort were excluded due to weak poor staining) and compared with scores manually generated by the pathologists. This too produced an excellent concordance with AUC = 0.81 and 0.79 (Figure 4C) utilizing the low-risk and high-risk cutoff TMEM scores (6 and 23, respectively).

## 3. Discussion

Though breast cancer mortality rates have declined in recent years due to better screening, the currently available prognostic markers cannot provide sufficient prognostic information regarding metastatic risk for all breast cancer patients; thus, overtreatment remains a significant challenge [24]. Preclinical studies have identified the mechanisms underlying hematogenous dissemination of tumor cells. These studies, which have utilized single-cell multiphoton imaging in live animals, a process known as intravital imaging [25,26], have identified microanatomical structures called the Tumor Micro-Environment of Metastasis (TMEM) which act as doorways through which motile tumor cells pass into the vasculature [11]. The TMEM doorway, composed of a stable interaction between a tumor cell which overexpresses the actin regulatory protein, Mena, in direct contact with a macrophage and a blood vessel endothelial cell, regulates junctional stability between adjacent endothelial cells and is the source of vascular leakiness and intravasation in tumors. 

Using this insight, prior studies have looked at the association between the number of TMEM doorways in patient breast cancer biopsies and excisions and in long-term metastatic outcome. These studies, conducted in three independent cohorts, have found that TMEM number is prognostic for increased risk of metastatic disease in early stage breast cancer (EBC), including ER-positive and HER2-negative disease [4,5,7]. TMEM number additionally provides prognostic information complementary to the Oncotype DX Recurrence Score (RS) 21-gene assay and IHC4 (a proxy for RS) [5,6].

Bringing a TMEM-based test into routine clinical use would thus provide clinicians with a significant tool to add to their armamentarium of biomarkers and would provide more accurate prognostic information in EBC; would reduce overtreatment and undertreatment; and in the neoadjuvant setting, would predict early in the course of treatment which patients are disseminating tumor cells in response to therapy [27].

The major limitation to moving the TMEM test into clinical use, however, has been the time required from pathologists for analysis of this marker. To address this and to demonstrate the analytical validity and reproducibility of the test, we developed an automated digital pathology-based algorithm (TMEM-DP) for scoring TMEM in breast cancer patient samples that have been stained with a multiplexed triple IHC protocol.

Our work shows that a multicelled morphometric-based digital pathology analysis can be developed which meets or exceeds current industry standards for analytical performance of in situ tissue-based diagnostic tests [28].

## 4. Materials and Methods

### 4.1. Cohort

The study was undertaken using a case-control study consisting of 259 case-control pairs, encompassing all clinical subtypes, that was nested in a cohort of 3760 breast cancer patients in the Kaiser Permanente Northwest (KPNW) health-care system [7]. As described by Rohan et. al. [7], these patients had received a first diagnosis of invasive ductal carcinoma of the breast between January 1, 1980 and December 31, 2000; were aged 21 years or older at initial diagnosis; were treated surgically; and did not have evidence of metastasis at initial diagnosis. From these pairs, 60 patient samples were chosen randomly and used for development of the algorithm which was then applied to the entire study population. All assays and analyses were performed blinded to patient outcome. The study utilized tumor specimens and clinical information from patients enrolled on trial E2197 (ClinicalTrials.gov identifier NCT00003519), coordinated by the Eastern Cooperative Oncology Group (ECOG).

### 4.2. IHC Triple Staining

As described previously [7], TMEM doorways are defined as the microanatomical sites of a Mena-overexpressing tumor cell in direct contact with a macrophage and an endothelial cell. As such, FFPE tissues are stained with a sequential triple immunostain for these cell types in the following order:

1. Endothelial cells—CD-31 (clone JC70A; 1:800 dilution; DAKO, Santa Clara, CA, USA) with Bond Epitope Retrieval Solution 2 and Vector Blue chromogen,
2. Macrophages—CD-68 (clone PG-M1; 1:300 dilution; DAKO) with antigen retrieval using Bond Epitope Retrieval Solution 1 and 3,3’-Diaminobenzidine (DAB) chromogen,
3. Tumor cells—anti-pan-Mena antibody (P/N: 610692, BD Biosciences, San Jose, CA, USA) that stain all isoforms of Mena, macrophages with Fast Red chromogen (Bond Polymer Refine Red Detection, Leica Biosystems, Buffalo Grove, IL, USA).

A light green counterstain was also applied to add normal tissue context. All staining was performed on the Bond Max Auto-stainer.

### 4.3. Manual TMEM Quantification

Manual quantification of TMEM was accomplished as previously published [4,7]. Briefly, slides were viewed and regions of high vascularity within tumor nests without artifacts such as retractions or folds were identified. Ten digital images were acquired at 400× total magnification and Red, Green, Blue (RGB) images were manually acquired and saved. TMEM assessment was performed using Adobe Photoshop Creative Suite 5. Opening each image, one at a time, the entire image was scored for TMEM doorways. One TMEM doorway is defined as a structure composed of an invasive Mena-overexpressing carcinoma cell (detected with pan-Mena, an antibody that recognizes all Mena isoforms), an endothelial cell, and a perivascular macrophage, all in direct contact and with no discernible stroma between the tumor cell and the perivascular macrophage. Using Photoshop, all TMEM were “marked” using a 60-µm diameter circle tool, and the “marked” images were saved as separate files. The total number of TMEM for each image was tabulated, and the scores from all ten images were then summed to give a final TMEM score for each patient sample, expressed as the number of TMEM per 10 high power fields (439 × 330 µm^2^ each). 

### 4.4. Digital Whole Slide Imaging

Slides were digitized for WSI analysis by the PerkinElmer Pannoramic 250 Flash II digital whole slide scanner using a 40× 0.95NA objective and a high-speed, 4 megapixel, color CMOS camera (pixel size = 0.12 µm) to acquire, stitch, and save an average of 13,757 fields in approximately 9.6 minutes occupying 7.5 GB for each slide. Dark-field intensity thresholding was used to automatically identify the tissue regions.

### 4.5. Statistical Analysis

All statistical analyses were performed in Graphpad Prism 7 (San Diego, CA, USA).

## 5. Conclusions

This validation study of the algorithm that we have developed demonstrates that TMEM-DP has high reproducibility, analytic performance, accuracy, and analytical precision and faithfully reproduces the pathologists’ workflow, all while reducing pathologists’ required time by an order of magnitude, thus enabling the TMEM score to be used in the clinical setting.

## 6. Future Directions

Validation and, now, automation of TMEM as a biomarker for metastasis set the stage for its use within the clinic. While, to date, TMEM doorway score has been associated with distant recurrence in only a subset of breast carcinomas (i.e., ER^+^HER2^−^) [5,7], TMEM doorways have been observed in preclinical models as well as clinical samples at the very early stages of breast cancer [29] as well as at metastatic sites such as the lung [30] and lymph nodes [31]. It remains to be determined if the TMEM doorways in these metastatic sites are active and responsible for the redissemination of tumor cells on to tertiary sites. Further, we are currently investigating the presence and function of TMEM doorways in other solid cancers such as pancreas and prostate as well as in Ewing’s sarcoma. If TMEM doorways in these other sites and cancers function with similar molecular pathways, it would present a unique opportunity to target dissemination across multiple cancer types. Finally, we are investigating how TMEM doorway score may be combined with other prognostic biomarkers (e.g., MenaCalc [32,33]) in order to expand its reach beyond the ER^+^HER2^−^ subtype.

## Figures and Tables

**Table 1:** Correlation of TMEM calculation metrics using Spearman rank.

Method of TMEM Quantification	Sum of TMEM-Associated Vessels	Sum of TMEM-Associated Macrophages	Total Interface Length between TMEM-Associated Vessels and TMEM-Associated Macrophages
Sum of TMEM-Associated Vessels	1.00	0.98 *p* < 0.0001	0.96 *p* < 0.0001
Sum of TMEM-Associated Macrophages	0.98 *p* < 0.0001	1.00	0.97 *p* < 0.0001
Total Interface Length between TMEM-Associated Vessels and TMEM-Associated Macrophages	0.96 *p* < 0.0001	0.97 *p* < 0.0001	1.00
